# Innovations for Reducing Methane Emissions in Livestock toward a Sustainable System: Analysis of Feed Additive Patents in Ruminants

**DOI:** 10.3390/ani12202760

**Published:** 2022-10-14

**Authors:** Valentina Caprarulo, Vera Ventura, Achille Amatucci, Giulia Ferronato, Gianni Gilioli

**Affiliations:** Department of Civil, Environmental, Architectural Engineering and Mathematics, Università Degli Studi di Brescia, 25121 Brescia, Italy

**Keywords:** greenhouse gas emissions, 3-NOP, probiotics, plant-based extracts, methanogenesis, rumen, dietary, compound feed, intellectual property

## Abstract

**Simple Summary:**

The mitigation of the environmental impact of animal production is a global objective, and innovation can provide new strategies and technologies to support the transition toward a more sustainable livestock system. Using patent data analysis to identify innovation dynamics, we explored the sector of feed additives to reduce methane emissions in ruminants. We found that this innovation sector is recent and rapidly expanding, with the European Union representing the center of innovation. The most promising inventions are related to the use of beneficial microorganisms (probiotics) and plant-based extracts.

**Abstract:**

An important challenge for livestock systems is the mitigation of environmental impacts while ensuring food security, and feed additives are considered as one of the most promising mitigation strategies. This study analyzed the innovation landscape of feed additives to reduce methane emissions in ruminants. The analysis is based on patent data to evaluate the development, scientific importance, and market-level impact of the innovations in this field. The results reveal that the EU is on the innovation frontier, with substantial and quality patent production. The innovation field is dominated by private players, characterized by high specificity in the R&D pipeline. Additives derived from plant or botanical extracts, together with 3-nitrooxypropanol (3-NOP), represent the emerging innovations, indicating a clear orientation toward more sustainable livestock systems. Despite the regulatory and semantic limitations related to the use of patent databases, data reveal a growing innovation activity at global level, which could lead to macroeconomic benefits for the entire livestock sector.

## 1. Introduction

Ruminant livestock plays an important role in satisfying the increased demand of high biological value protein, as well as in terms of food safety and security, which could lead to improvements in human health and preservation of the environment [1]. Animal products, such as meat, milk, and eggs, are a source of essential nutrients such as heme-iron, vitamin B12, vitamin D3, zinc, calcium, and high-biological-value proteins characterized by high digestibility, containing all essential amino acids [2]. However, one-third of the total CO_2_eq emissions are represented by livestock sector, which account for 14.5% of total anthropogenic emissions [3]. In general, the main emission originated from agricultural sector is related to some specific greenhouse gases (GHGs) such as methane (CH_4_), nitrous oxide, and carbon dioxide [2]. Thus, the reduction in ruminants’ enteric CH_4_, which is considered the most prevalent enteric emission in ruminants, is considered the most effective enhancement target in reducing global warming in the short term due to a lower atmospheric residence time value and greater power in heating the atmosphere than carbon dioxide [4]. Therefore, several mitigation strategies for reducing enteric CH_4_ have been suggested, and a relevant number of extensive reviews are available on this issue [1,5,6], in which animals’ diet manipulations play a pivotal role [1,5]. CH_4_ mitigation strategies could embrace different diet formulation approaches, e.g., adopting specific feed ingredients in animals’ diets such as protein, lipid, and forage–concentrate ratio, or supplementing specific dietary feed additives able to directly inhibit methanogens or alter metabolic pathways leading to reduced substrate for methanogenesis [7,8]. Furthermore, the presence of clear market drivers and the development of effective regulatory system are key factors for the global achievement of mitigation goals through innovation.

However, to our best knowledge, there is a lack of studies dealing with the innovation landscape of feed additive for reducing CH_4_ emissions in ruminants. In this context, the main aim of this work was understanding the worldwide innovative landscape of feed additives for reducing CH_4_ emissions in ruminants. More specifically, the goal was to evaluate the development, scientific importance, and market-level impact of anti-methanogenic feed additives. Various strategies are adopted to measure innovation. Among the existent indicators, research and development (R&D) expenditures and the number of scientific personnel employed full-time are the most widely used [9]. Although these indicators provide a comprehensive view of the overall innovation system, there are some drawbacks in their use for measuring innovation with environmental targets. For example, indicators based on R&D expenditures measure inputs to the innovation process, whereas a measure of outputs of innovation would be preferable [10].

To mitigate these limitations, there are two types of innovation output indicators that can be analyzed: bibliometric data (scientific publications) and technometric data (patent publications). Patent data are considered a standardized and objective source that is a good proxy for measuring innovation activities [11]. Patent data are best suited to identify specifically environmental innovation because patent classification systems are by their nature technological and allow technologies to be characterized [12].

## 2. Materials and Methods

The research methodology is based on the extraction and processing of patent data. Data extraction is structured on the formulation of a search query using Questel’s Orbit Intelligence database, an intellectual property business intelligence software dedicated to the search and analysis of patent documents with global coverage. The identification of the specific area of innovation was achieved through the selection of an appropriate combination of keywords [11]. The search interface of the software allows for a selection of keywords associated with the field of investigation and contained in the patent documents; to target the search effectively, word operators were used, and keywords were combined using so-called “Boolean operators” such as “AND” and “OR”. It was also chosen not to circumscribe the field of study by including the International Patent Classification (IPC) and Cooperative Patent Classification (CPC) codes in the query. Although this approach contains the risk of potentially including the wrong patents with too loose a formulation, the use of IPC/CPC codes leads to an overly specific resolution of the field of survey, preventing the cross-cutting nature of environmental technologies from being captured [13].

The final query structure entered the Questel IP Orbit Intelligence platform in April 2022 for data extraction was as follows: ((CATTLE OR LIVESTOCK OR RUMINAN+) AND (FEED OR DIET+ OR ADDITIV+) AND (METHAN+ OR EMISSION OR MITIGATION OR CLIMATE CHANGE OR NITROGEN EFFICIENCY)).

Here, “+” stands for including all possible words beginning with the previous term. The keyword combination was used to search the following fields in the patent document: title, abstract, keywords in context, claims, description, and object of the invention.

The raw patent data consisted of 916 patent families corresponding to 8877 individual patents. A patent is a legal title that confers to the holder a temporary monopoly for the commercial exploitation of an invention [14]. Patent family relates to a set of patents of the same invention, granted anywhere in the world (usually the first publication is in the assignee’s domestic patent office); conversely, the term patent regards the single patent document.

In a first step, a qualitative analysis of the patent families available in the bulk dataset was performed. Patent families are distinguished by a common patent family ID. The purpose of this analysis was to initially distinguish the different patent families on the basis of their legal state, to enable a comprehensive analysis of the innovation process by including all inventions, and then discriminate among those for which players have retained an interest in intellectual property protection [15]. The qualitative analysis then focused on introducing a technical classification criterion not provided by the Orbit Intelligence system: “specific target of the invention” which made it possible to eliminate those patent families whose target audience did not include the specific focus of the research; “functional group”, a classification that can associate the functional groups to which the technology belongs based on the European Food Safety Authority (EFSA) classification (Reg. 8131/03), thus allowing the dataset to be aligned with the regulatory system; “additive type”, specification regarding the type of patented technology associated with the individual patent family IDs. The analysis was conducted by evaluating “title”, “abstract”, “claims”, “object of the invention”, “technical concepts”, and “advantages published” in patent document reports. The information provided by CPC/IPC codes provides an initial reference for identifying technologies in a specific technical field, but it is not sufficient [12]. The use of such data did not easily allow for the specific identification of the technologies of interest, generating the needed to reconstruct these aggregates on the basis of specific knowledge and a clear and operational definition of the technical field of interest [9].

This step in the selection process involved evaluation by two independent researchers, and their coding results were compared through inter-coder agreement, which ensures the validity of the research results. In our study, the agreement between the two coders (Cohen’s kappa) [16] was 97.8%, and the remaining differences were resolved through personal consultation between the two coders. The evaluation caused the exclusion of 803 patent families corresponding to 8447 patents (not relevant to the field), leaving 113 patent families and 430 patents for the next stage of the analysis.

Patent data were combined with non-patent scientific literature (NPL) for the investigation of the interaction between the advancement of scientific knowledge and the technologies, representing a proxy for the scientific importance of the invention, as well as the quality of the patent [12].

The elaboration of patent data was then performed to identify and analyze the inventive process, characteristics of technologies, and market impact of patent strategies (Table 1).

Technologies were examined in terms of family size, proximity to science, and nature of technologies to identify specific environmental aspects. The combination of the variables in Table 1 enabled the construction of indicators. The average age of a patent family by country was obtained, e.g., from the difference of the last publication date and the earliest priority date in a single family, divided by the number of total families belonging to the country. The same indicator was calculated by the type of additive. The variable “average patents per family” was constructed both by country and by single assignee, as the total count of patents divided by the number of patent families.

## 3. Results and Discussion

### 3.1. The Dynamics of the Inventive Process

**Figure 1:**
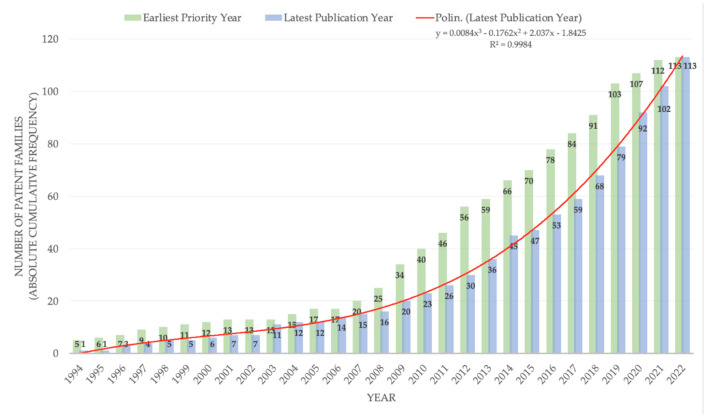
Temporal development trend of the innovation process.

Time trend analysis of the innovation process revealed that, until the end of the first decade of the millennium, patent activity related to feed additives for mitigating ruminants’ CH_4_ emissions was limited. According to the earliest priority year (year of the first filing of the patent application as a reference for the earliest date of invention), the total number of patent families worldwide was 19 (16.8% of the analyzed portfolio) until 2007 (Figure 1). However, from the following year, there was an increase in the number of filings, with an annual average of 6.2 patent families.

The growth dynamic is even more evident when considering the latest publication year (year in which the patent application was last published for a specific patent family), which showed a marked increase over the years, with the trend described by a second-degree polynomial function (Figure 1).

Over the past 5 years, the publications increased by 409.7% compared to the entire previous period, suggesting that a *patent race* is still ongoing. In this context, the *patent race* occurs when companies compete to produce a specific invention, indicating that the technology has reached its own maturity in inventive activity. In addition, the patent race itself acts as an incentive for innovation, generating a growth forecast for intellectual property related to feed additives able to reduce CH_4_ emissions from ruminants. The sector maturity could also be interpreted by analyzing the legal status of the patent portfolio. The information derived from the legal status could lead to better discrimination of patent families characterized by at least one member with a grant, and families under examination. In addition, the information from the legal status of a patent could be useful to evaluate the percentage of patents that are no longer in force, such as lapsed, revoked, or expired. Our data showed that 31% of the families have completed the patent granting process, and 22% are under evaluation, despite having already been made public, while as many as 23% are lapsed, indicating the high selectivity of the sector and consequent abandonment of less profitable technologies.

The decrease in the variable earliest priority year for 2022 can be attributed to the time lag (18–24 months on average) between the patent application and its publication in the patent databases.

**Figure 2:**
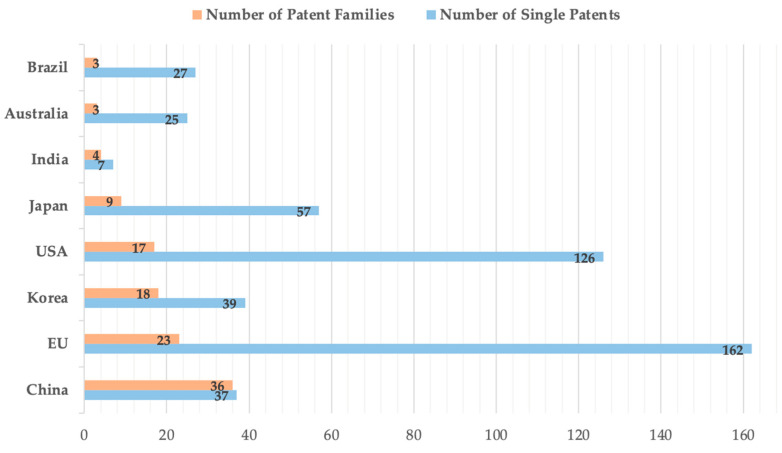
Number of patent families and individual patents by priority country.

The analysis of the geographical distribution of inventions (Figure 2) revealed that Europe (or more precisely the European region, including countries that are not officially EU members while affiliated with the European Patent Office, such as Switzerland and the United Kingdom) was the second world’s leader in the production of innovation in the field. EU inventions (patent families = 23) represented 20.5% of the total, with growing relevance in the count of individual patents (*n* = 162, 33.8% of all patent activity).

Although representing an aggregate figure that includes the inventive activity of different states, it is a significant indicator of a specific aptitude of the European area to invest in research for this sector [17]. Worldwide, the top four players own 83% of inventions (China, EU, Korea, and the United States), indicating a moderate polarization and that the inventive process in some worlds’ areas has yet to take hold. A lower share of patent families was detected for Japan, India, Brazil, and Australia (nine, four, three, and three, respectively).

**Figure 3:**
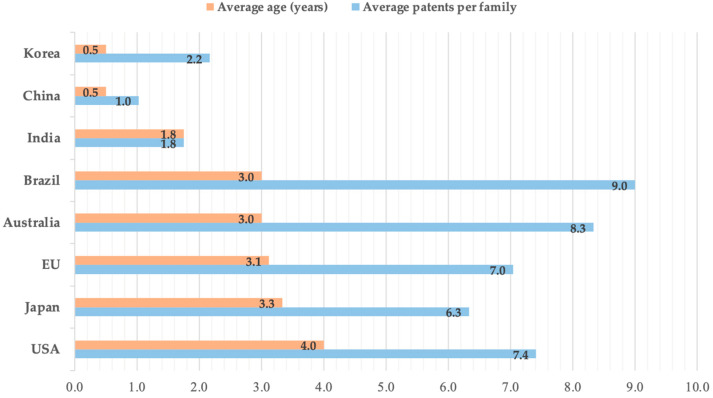
Average age of patent families and average number of patents per family.

Data from the average age of patent families and average single patents per family (Figure 3) could give important information regarding the area in which the inventions were initially developed and the diffusion of the inventions [9]. The analysis indicated that the United States, Japan, Europe, Australia, and Brazil with an average age of 4, 3.3, 3.1, 3, and 3 years, respectively, were the pioneers in research on feed additives for reducing CH_4_ emission in ruminants. In addition, the data showed the recent development of research in the field, with an average age of 3.28 years aggregating the first five countries. The average value of the number of patents, from which each family is composed, indicated the tendency of actors in each country to spread the invention to more than one patent system [12]. A higher average value of patents/family indicates a greater ability to spread innovation [14]. Thus, it was found that China, the leader in the number of patent families, shows a much lower value (on average, 1.0 patent per family) than Europe and the United States (7.0 and 7.4, respectively). This result represented how inventions developed in China tend to be registered only in the national patent system. In contrast, Brazil and Australia emerged as systems where inventive activity is not among the most prolific, but the dissemination of inventions is extremely effective (average of 9.0 patents per family for the former and 8.3 for the latter).

### 3.2. Types of Innovation: Characteristics and Specificity of Inventions

**Figure 4:**
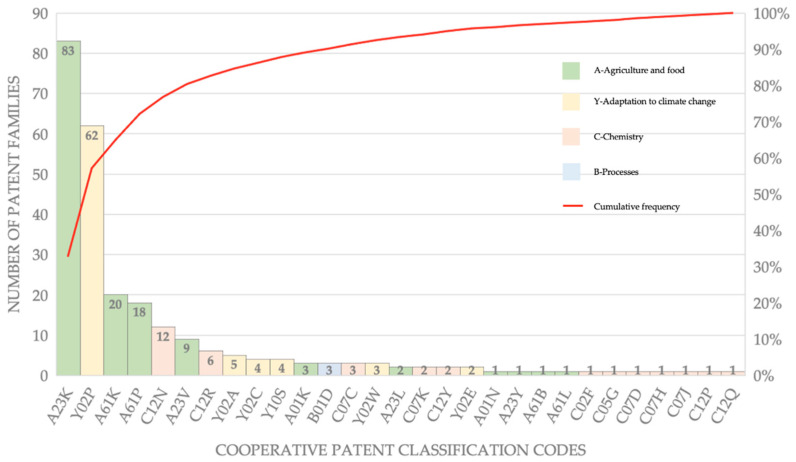
Patent families by CPC codes and relative weight in the portfolio.

An important focus was on the type of innovation, which was initially assessed by extracting and classifying the CPC codes associated with individual patent families (Figure 4).

The data first confirm the validity of the analyzed portfolio, with the codes A23K (fodder) and Y02P (climate change mitigation technologies in the production or processing of goods) having a relative weight greater than 60%. More than 80% of inventions were characterized by including codes A61K (preparations for medical purposes), A61P (specific therapeutic activity of chemical components or medicinal preparations), and C12N (microorganisms or enzymes).

The classification made during the qualitative analysis and based on the functional groups indicated by EFSA allowed distinguishing individual patents by type of additive (Table 2).

Data for the types of feed additives showed that the innovation process involved many application fields, with as many as 113 patent families and an average of only 4.2 patents per family. Thus, our results showed that probiotics represented the first technology with the highest value for both patents and patent families [9]. This is indicative of the cross-cutting nature of the specific field of research, in which different types of microbial strains are tested and subject to intellectual property protection with an average patent per family of 2.8. The FAO/WHO 2002 defines probiotics as live microorganisms able to confer a health benefit on the host when administered in adequate amounts [18]. Thus, this is referred to as direct in-feed microbials (DFMs), basically live microorganisms that, when administered in adequate amounts, have health benefits for the animal. In particular, specific probiotics adopted as DFM include several bacterial and fungal species such as *Bacillus*, *Bifidobacterium*, *Enterococcus*, *Lactobacillus*, *Propionibacterium*, *Megasphaera elsdenii*, *Prevotella bryantii*, *Aspergillus*, and *Saccharomyces* [19]. However, lactic acid bacteria including *Lactobacillus*, *Bifidobacterium*, and *Streptococcus*, are widely used as probiotic strains in the supplementation of ruminant feed [19].

The scientific community is still largely studying the effect of probiotics administration in ruminants due to the prominent effect on rumen microbial ecosystem, feed digestibility, and degradability when administered in adequate amounts. Therefore, the selection of specific bacteria strains could mitigate climate-altering gas emissions from ruminants by acting through competitive inhibition of methanogenic microorganisms in the rumen. In addition, probiotics are also able to improve the bioavailability of microbes, as well as digestive capacity, and reduce ruminal pH and lactate levels [20]. The beneficial effects of probiotic supplementation in ruminants, especially in growing animals, is also related to the possibility of replacing antibiotic with prebiotics due to their effects for protecting gut microbiota [20].

Several reviews on the use of probiotic as CH_4_ mitigation strategies have been published over the years; however, the research on this topic in ruminants is still controversial due to inconsistent results among studies [20,21].

A recent meta-analysis published by Darabighane et al. (2019) [22] on the efficacy of a specific yeast, *Saccharomyces cerevisiae*, on methane production in dairy and beef cattle showed discordant results. In particular, the study concluded that the supplemented yeast to ruminants did not have significant effects in terms of a reduction in CH_4_ production. The authors concluded that many factors could influence the yeast effects for reducing CH_4_ production, such as the small number of studies included, the technique adopted for measuring the CH_4_ (sulfur hexafluoride method or respiratory chamber techniques), management factors, climate, and physiological stage of the animal. Therefore, further research studies are needed to better understand the effects of probiotics on reducing CH_4_ production in ruminants.

The second technology by the number of patents is cashew nut shell liquid (CNSL); however, this type of additive belongs to a single patent family, a sign of a specific and well-circumscribed field of investigation, as also described by the average value of patents per family (*n* = 31). CNSL is a byproduct of the cashew nut industry with several industrial applications (producing paints, lacquers, coatings, brake linings, and others) and is also adopted as feed or feed additive in livestock. CNSL acts in ruminal fermentation by regulating the microbial community, improving rumen fermentation, and reducing CH_4_ emissions [23]. In vitro and in vivo studies showed considerable effects of CNSL in reducing CH_4_ emissions and increasing propionate production in the rumen [23].

The third type of innovation by patent quantity concerns heterogeneous formulations (feed formula), which represent and involve multiple patent families, although in this case it is probably due to the inherent low specificity nature of the inventions (average patents per family = 3.5). The technology with the second highest value of average patents per family (*n* = 24) is flavanone glycoside. Flavonoids, such as flavanone glycoside, represent an important class of polyphenols with beneficial effects for their antimicrobial, antioxidant, radical-scavenging, and anti-inflammatory activities, as well as beneficial effects on the immune status. In ruminants, flavonoid supplementation showed an interesting ability to reduce methane production, either indirectly or via direct action against methanogens. Thus, the reduction in or selection of specific Gram-positive bacteria could increase the production of propionate compare to acetate; these manipulations can both contribute to increased efficiency of the ruminant animal [24], improve volatile fatty acid synthesis, and reduce rumen ammonia concentration and methane production by rumen [24,25]. In assessing the NPL average references per patent family, it is technologies related to enzymes (*n* = 130) and probiotics (*n* = 75) that have the greatest significance. This identifies how it is the field related to the microbiological field of investigation that generates the most innovations aligned with scientific research. The innovative players in these areas turn out to be the players that most collaborate with public research institutions, such as universities.

**Figure 5:**
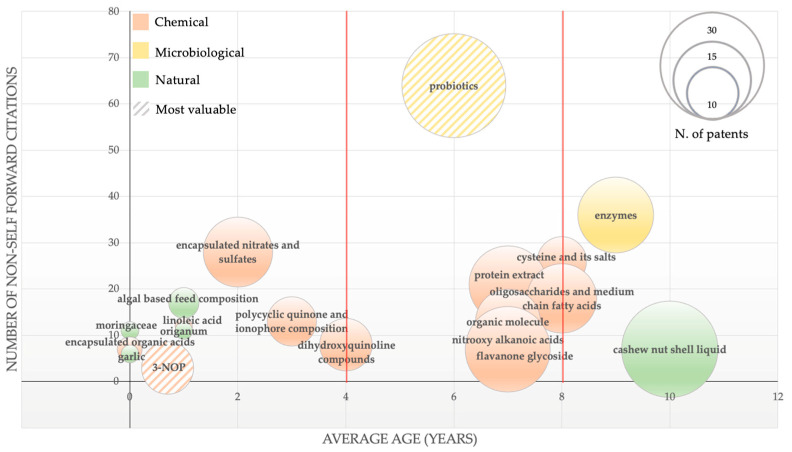
Emerging, pioneering, and disruptive technologies (>5 non-self-forward citations).

However, this type of analysis represents a quantitative assessment of innovations in the field. A more in-depth picture is provided in Figure 5 with the qualitative analysis of patents classified by additive type. In detail, information on the number of patents (represented by the size of the spheres), the average age of the patent families, and the number of non-self-forward citations (which indicates how much an invention has been cited in subsequent patents and is considered a proxy for the value of the invention itself) was combined.

The result is a scenario in which three main groups of innovations can be identified: technologies with an average patent age of more than 8 years, consisting of the use of enzymes and CNSL, which represent the pioneering fields of investigation; the second group of technologies with an average age between 4 and 8 years, where the highest average citation values and number of patents are located, such as nitrooxy alkanoic acids, flavanone glycoside, oligosaccharides, and medium-chain fatty acids (in this group, the technologies regarding probiotics can be defined as disruptive invention and emerged with prominence); the third group of emerging technologies, consisting of different fields of investigation that have entered this specific area of innovation in the last 4 years, is indicative of the significant potential for development. Among these encapsulated nitrates and sulfates, 3-NOP, plant extract (Moringacae, garlic, and Origanum, among others) and algal-based feed composition are qualitatively the most relevant technologies.

Several in vivo studies reported beneficial effects of encapsulate nitrates and sulfates due to the ability of nitrate to compete with methanogens for hydrogen utilization and acting as an alternative hydrogen sink in the rumen of beef steers, dairy cows, sheep, and goats [26]. Regarding the supplementation of 3-NOP, increasing research and meta-analyses support the beneficial effect of 3-NOP for reducing CH_4_ emissions from ruminants [27]. 3-NOP is a chemical compound able to reduce CH_4_ emissions due to its structural similarity to methyl coenzyme-M, which inhibits the activity of methyl coenzyme-M reductase related to the final step of methanogenesis [26]. Another group of emerging additives is represented by plant extracts. Plants extracts are a heterogeneous group of natural alternative to chemical additives, such as Moringacae, garlic, and Origanum, with the ability to reduce CH_4_ [28].

A further trend emerges for inventions with an average age of 2 years; despite a low number of citations (a physiological aspect related to the limited time of publication), an already significant patent production is evident for those technologies referring to natural and botanical extracts, a field of innovation with the highest momentum and scope for development.

Currently, no studies have deeply analyzed the patent landscape of feed additives for reducing GHG emissions in ruminants. However, worldwide several surveys have been conducted for evaluating feed industry or industry preparedness, in terms of understanding feed industry market, trends, and technology of feed industry worldwide [29,30,31,32].

In general, feed manufacturing advanced rapidly from the beginning of the 20th century, showing its consolidation worldwide over the years with a more intensification of the production and the adopted technology [30]. Despite the technologies adopted to improve the qualitative and quantitative production of feed, the feed industry is faced with another emerging issue related to improving animal performance characteristics, minimizing costs, and maximizing feed production efficiencies. If considering the worldwide context, particularly the European one, a global effort has been undertaken to achieve the main goals of sustainability. Indeed, after decades of work by countries and the United Nations, 17 Sustainable Development Goals have been developed as part of the 2030 Agenda for Sustainable Development. In this context, the European Union made a positive and constructive contribution to the development of the 2030 Agenda. This scenario is touching all compartments of production related to the agricultural sector and consequently also feed industry production. Indeed, in line with this global challenge, the feed industry also considers animal health, animal efficiency and productivity, environmental protection, food safety, and food quality as the most important aspects which allow producing sustainable animal products [30,33].

A survey study conducted by Caprarulo et al. (2016) [31], aimed at evaluating the potential research and development tendency in the Italian and Serbian feed industry, showed that they adopted specific groups of additives, such as antioxidants, enzymes, probiotics, and flavoring. In line with our results, a study conducted by Caprarulo et al. (2016) [31] investigated the potential areas for research and development in the feed sectors, showing that the most adopted types of feed additive in the feed industry were superimposable for enzymes and probiotics. In addition, the same study showed that the feed industry uses feed additives with the aim of reducing animal emissions. Hegarty et al. (2021) [32] conducted a survey aimed at evaluating the industry preparedness for methane-lowering feed additives. The survey by Hegarty et al. (2021) [32] showed that the only two of 14 responders produce feed product with a low methane claim. In addition, only 14% of responders considered the use of feed or supplements to reduce enteric emissions as a high or extreme priority, while 43% of responders are expecting to modulate this priority within 5 years. In the same study, the most well-known feed additives for reducing methane emission were probiotics (64%), essential oils (50%), and antibiotic rumen modifiers (50%). On the other hand, the responders from the same survey were only aware of 3-NOP, Asparagopsis (algae), and nitrate as additives for reducing methane emission. However, more than 60% of survey responses were using probiotics and 50% antibiotic rumen modifiers as feed additives, primarily due to increased animal performance (81%), with improved feed efficiency (73%) and health (69%) being the next most important.

### 3.3. Key Players in Innovation and Target Markets

The analysis of assignees revealed that 113 patent families are owned by 76 different entities, both public and private (Table 3). These data provide important insights into the characteristics of the innovation sector under consideration, which was found to be of recent development and composed of a multiplicity of individual actors of different natures and backgrounds.

For the analysis, major players (at least five patents or at least two patent families) were considered first.

The CR_4_ concentration index, the concentration ratio for the top four assignees [34], was found to be 0.34 by single patents, while the CR_10_ index (concentration ratio for the top 10 assignees) reached 0.56. Each actor was found to own, on average, 6.3 patents, even though 36 assignees out of the total of 76 (47.3%) owned only one. Technological change may potentially have been a driver of this concentration trend. It has often been proposed as an explanation for the increase in market share of leading companies [35].

The data showed the presence of one entity in a predominant position, the Dutch company DSM, with the highest number of inventions (*n* of patent families = 12) and individual patents (*n* = 86). Although the average age of the patent families was rather low (2.9 years), the high number of citations received (*n* = 31) made it possible to identify the significant role of this company’s research and innovations. Forward citations were used to assess the technological impact of inventions and the economic importance of patents. The value of a patent and the number of its citations were found to be repeatedly correlated [12]. The second player in terms of number of patent families was found to be the Chinese public Feed Research Institute Sciences, albeit with a low production of individual patents (*n* = 4). This resulted in an average number of patents per family of one. In addition, a low number of non-self-forward citations were found (*n* = 3), indicating a low innovative production and whose diffusion was limited to the national context only. The third assignee by the number of patent families, was found to be Locus, a private US company (*n* of patent families = 3; *n* of patents = 21). The average age of families for this company also emerged relatively low (1.3 years), confirming the recent development of the field under analysis. A number of 19 non-self forward citations and an average of seven patents per family described a quality patent production, capable of spreading to the most important markets and serving as a benchmark for the innovative development of the industry [12]. While DSM has focused its R&D efforts on several application areas, with a recent predominance of 3-NOP technology, Locus is a company with extensive knowledge of microbial applications and biosurfactants, emerging as the innovative leader in the introduction of probiotics. Specifically, Locus Animal Nutrition (Locus AN), an operating division of Locus Fermentation Solutions (Locus FS), has identified, tested, and produced non-genetically modified and microbial direct feeding feed additives for animal production systems. The recent focus on cow emissions led Rabobank, a Dutch multinational banking and financial services company focused on the food and agribusiness sector, to name Locus AN among the top 15 AgTech startups to develop solutions for a sustainable food system in 2020. Another relevant innovative player turned out to be the private Brazilian company GRASP Industria & Comercio, with quality research on encapsulated nitrate and sulfate technology with two patent families, 16 single patents, and 28 non-self forward citations.

The public institutions that emerged in the list of top players belong mainly to the Asian public system, predominantly Chinese and Korean. Indeed, these systems have appeared to base their R&D on public funding, with the institution itself then owning the invention. The data showed, however, a low quality of inventions (low values of non-self-forward citations) and a consequent difficulty of development outside national borders (low values of average patents per family). Focusing on qualitative parameters, the public entities found to be most influential were the Commonwealth Scientific and Industrial Research Organization (CSIRO), an active Australian government research organization, and the Cornell Research Foundation, an example of a public/private institution referring to the US Cornell University. It is the latter, when analyzing the number of non-self-forward citations (*n* = 27), that was found to have a higher specific weight in terms of research quality. The Australian institution was found to be focused on the field of marine red microalgae as an additive feed for ruminants to reduce emissions, while Cornell University and its foundation protected inventions related to microalgae and enzymes.

**Figure 6:**
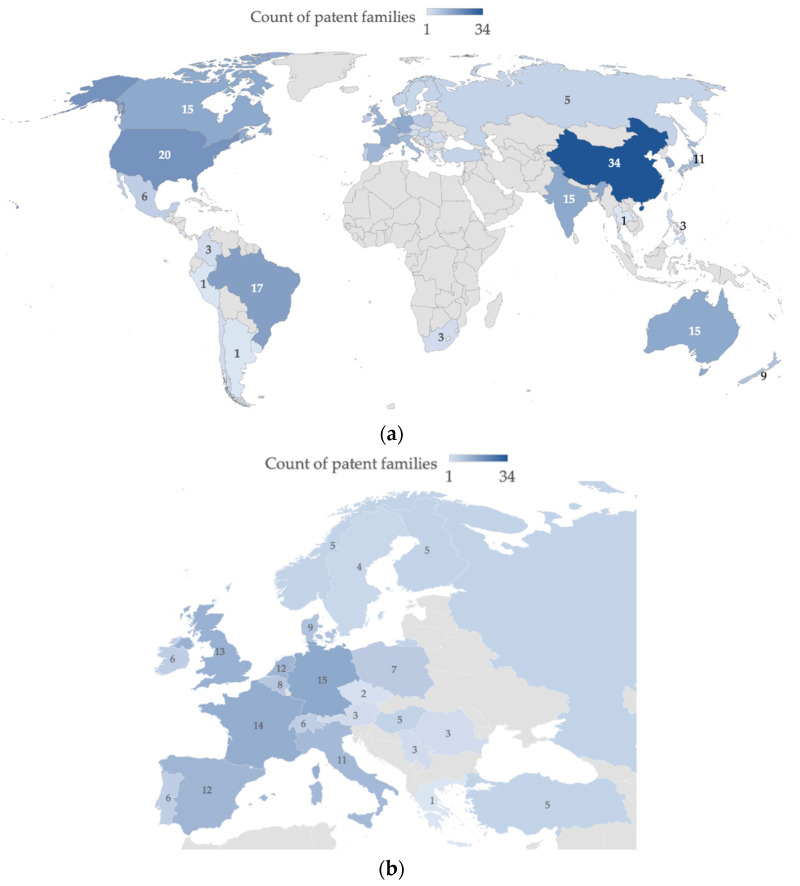
Count of patent families by protection country: (**a**) global scenario; (**b**) focus on European region.

An additional level of detail involved the analysis of the publication strategies of innovative players in the industry. The protection country variable was considered, which provided information on the countries in which each patent was registered, identifying the major markets [12] (Figure 6).

The data confirmed that Europe represents the center of patent activity, not only in terms of the origin of inventions (Figure 2), but also as a patent system capable of attracting patents of foreign origin; a total of 63 patent families were registered in the European region. Considering that the number of patent families originating from EU subjects amounts to 23, it is shown that nearly 63.5% of the patent families registered in Europe are derived from non-EU subjects. Emblematic cases may be Spain and Italy, where the near absence of innovative players did not prevent national patent systems from registering 12 and 11 patent families, respectively. Similar dynamics emerged for non-EU countries such as Canada, Brazil, India, and Australia, where interest in industry-related intellectual property protection was high, a strong indication of a significant market for all players [12].

Data provided by members of the European Feed Manufacturers’ Federation (FEFAC) confirmed that Europe dominated the compound feed market with 150.2 tons in 2020, up 1.3% from 2019. EU-27 compound feed production accounted for 13% of global production, estimated around 1172 tons, up 5.5% from 2018. However, the EU’s global market share decreased by 2% in 2020, mainly due to the increase in feed production in Asia over the past decade [36]. In accordance with the findings, southern China is a massive producer of animal feed and, as a result, has a huge market for the import of feed ingredients. Guangdong province alone is the largest feed-producing province in all of China, with 28 million tons in 2016, up 10% from the previous year. For 2017, industry analysts estimate that feed production in this province will rise to 30 million metric tons [37]. For the North American region, the USDA has estimated that the feed market will grow at a compound annual growth rate of 2.84% through 2028. The demand for meat products is expected to increase, with Canada as the main developing market, as found in our analysis. Canada has a high demand for feed and imposes a lower cost, which will become significant for increasing market sales [38].

Lastly, FEFAC data showed how feed costs have increased more than producer prices over the past 25 years in the EU, confirming a general trend of permanent pressure on farmers. With feed being the most important cost factor in livestock production, accounting for up to 14% of the farm value of cattle in 2020 [36], this explains the need for Europe to be on the innovation frontier and for non-EU countries to protect their inventions in the European region and gain market share.

## 4. Conclusions

The study provided an overview of current trends in the patenting of feed additives for emission reduction in ruminants. Several patent indicators were used to provide insights into the technological maturity of the industry and the positioning of different countries and players in this innovation process. Patent activity was found to have a marked increase globally, and, although with different strategies, all the countries analyzed are addressing the need for a transition to a new way of reconciling the livestock industry with GHG mitigation policies. The European region, considered a benchmark for the feed industry, showed in this assessment a patenting activity strongly stimulated by the common political system. The results hypothesize positive macroeconomic impacts for feed additive manufacturers, such as increased growth, investment in new additives, innovation, research, and exports of new and innovative feed additives. The microeconomic impact on small and medium-sized enterprises should be specifically assessed, including the increase in innovation potential that could involve them. Their involvement in the innovation system appears crucial to enable the development of technologies that can be integrated into business practice. Currently, R&D activities are effectively confined to rigid systems, supported in several countries by strong public and private actors. The impact on the production chain cannot yet be properly assessed until farmers have access to appropriate tools to adapt technologies. In this context, policy implications assume significant specific weight. European policies stimulate research and, thus, patenting activity, demonstrating the effectiveness of its strategic orientation.

From a technological perspective, the strongest growth trend is led by the plant-based additives sector. The increased attention on environmental issues and the need to redeem an entire production system, too often identified as the only responsible for climate change, probably oriented R&D efforts on this field, generating the basis for a sustainable future for the sector.

However, it is important to report the limitations associated with the use of patents to develop a comprehensive measure of innovation. Indeed, not all innovations are patentable. Patents are designed to protect only technological innovations that meet the patentability criteria by limiting the nature of innovations that can be measured. Moreover, not all patentable inventions are patented, and alternative intellectual property management strategies (trade secret) can make the data incomplete, or patent statistics can be biased. Lastly, patented inventions vary in quality and maintenance costs, generating bias in quantitative assessments of an industry’s innovations. Nevertheless, patent data are globally quoted as suitable indicators for describing economic developments, as well as international relations among the economy, society, and technology, a crucial aspect in environmental impact mitigation.

The scenario seems complex; our data showed that innovation is deeply following the global scenario to perceive a sustainable way according to the global decisions to reduce the environmental impact of animal production. Nevertheless, more efforts are needed to increase the connection among scientists, feed industry, and feed additive industry. Thus, more studies are needed to better understand a common way to develop feed additives or feed able to effectively reduce methane emissions from ruminants.

## Figures and Tables

**Table 1:** Patent variables and their use in the analysis.

Area of Investigation	Variables	Description of Variables	Role in the Analysis
Inventive process	Earliest priority year	Year of the first filing	Development over time
	Latest publication year	Year of the last publication	
	Earliest priority country	Country of the first filing	Where the invention originated and which countries were most prolific
	Patent families	Number of patent families	
	Single patents	Number of individual patents	
	Earliestpriority date	Date of the first filing	
	Latest publication date	Date of the last publication	
Characteristics of technologies	CPC ^1^ codes	Classification by technology field	Relative weighting of the technology areas
	Patent families	Number of patent families	
	Type of additive	Classification criteria adopted in line with EFSA ^2^ guidelines	Identification of technologies
	Non-self-forward citations	Received citations in other patents by type of additive	Technological impact of an invention
	Average age	(latest publication date—earliest priority date)/number of patent families	
	Single patents	Number of individual patents	
	NPL ^3^ average references	Average citations per patent in NPL	Interaction between scientific knowledge and technologies
Key players and main markets	Standardized assignee	Patent owners grouped with standardized algorithm	Identification of key players
	Current assignee	Current patent holder	
	Average patents per family	Average number of patents per family byassignee	Technology diffusion index
	Non-self-forward citations	Received citations in other patents by assignee	Quality index of R&D ^4^ of an assignee
	Protection country	Patent office chosen to protect the invention	Target markets

Abbreviations used: ^1^ Cooperative Patent Classification; ^2^ European Food Safety Authority; ^3^ non-patent literature; ^4^ research and development.

**Table 2:** Main types of additives and relative patent production (>5 patents).

Type of Additive	Number of Patents	Number of Patent Families	Average Patents Per Family	Non-Patent Literature Average References
Probiotics	36	13	2.8	75
Cashew nut shell liquid	31	1	31.0	8
Feed formula	27	7	3.9	24
Flavanone glycoside	24	1	24.0	19
Nitrate and sulfate	23	1	23.0	12
Protein extract	20	1	20.0	14
Enzymes	19	2	9.5	130
Red marine macroalgae	18	1	18.0	6
Encapsulated nitrates and sulfates	16	2	8.0	28
Oligosaccharides and medium-chain fatty acids	16	1	16.0	25
Lauric acid and 3-nitrooxypropanol	15	1	15.0	12
Nitrooxy organic molecules	15	1	15.0	24
Organic molecule	14	1	14.0	10
Rumen protected non-protein *n*	13	1	13.0	17
Method	12	3	4.0	16
Para nitro amino derivates	12	1	12.0	16
Nitrooxy alkanoic acids	11	1	11.0	13
3-nitrooxypropanol	9	4	2.3	13
Diallyl disulfide, nitrate and eucalyptus oil	9	1	9.0	13
Dihydroxyquinoline compounds	9	1	9.0	30
Pasture treatments (beneficial microorganism)	9	1	9.0	5
Cysteine and its salts	8	1	8.0	4
Polycyclic quinone and ionophore composition	8	1	8.0	5
Eugenol; cinnamaldehyde; extract of a plant belonging to the alliaceous family	7	1	7.0	9
Nitroaniline derivative or a salt	7	1	7.0	13
Lignin	6	1	6.0	6
Prebiotics	6	1	6.0	3
Total	480	113	4.2	6.8

**Table 3:** Analysis of major assignees (>5 patents or >2 families).

Assignee	Nation	Private/Public	Number of Families	Number of Patents	Average Patents Per Family	Family Average Age	Non-Self Forward Citations
DSM IP Assets	Netherlands	Private	12	86	7.2	2.9	31
Feed Research Institute—CAAS	China	Public	4	4	1.0	1.5	3
Locus IP	USA	Private	3	21	7.0	1.3	19
Institute of Animal Sciences—CAAS	China	Public	3	3	1.0	0.0	0
Grasp Indústria & Comércio	Brazil	Private	2	16	8.0	2.0	28
Ajinomoto	Japan	Private	2	6	3.0	2.0	4
Blackcarbon	Denmark	Private	2	4	2.0	1.0	3
Agricultural University of Hebei	China	Public	2	2	1.0	1.0	0
China Agricultural University	China	Public	2	2	1.0	3.0	10
Indian Council of Agricultural Research	India	Public	2	2	1.0	1.5	0
Korea National Open University/Industry Academic Cooperation Foundation	Korea	Private/public	2	2	1.0	0.0	1
National University Chonbuk	Korea	Public	2	2	1.0	0.0	3
Sichuan Agricultural University	China	Public	2	2	1.0	0.5	6
Zhejiang University	China	Public	2	2	1.0	0.0	3
Idemitsu Kosan	Japan	Private	1	31	31.0	10.0	7
Healthtech Bio Actives U	Spain	Private	1	24	24.0	7.0	7
Cargill	USA	Private	1	23	23.0	10.0	18
Alltech	USA	Private	1	20	20.0	7.0	21
CSIRO	Australia	Public	1	18	18.0	7.0	3
Nutrition Sciences	Belgium	Private	1	16	16.0	8.0	18
Cornell Research Foundation	USA	Private/public	1	14	14.0	15.0	27
Evonik Operations	Germany	Private	1	13	13.0	5.0	2
Merck Sharp & Dohme	USA	Private	1	13	13.0	10.0	23
C Lock	USA	Private	1	10	10.0	7.0	48
Ableco Finance	USA	Private	1	9	9.0	4.0	8
Alberto Samaia Neto	Brazil	Private	1	9	9.0	4.0	2
CJ Feed & Care	Korea	Private	1	9	9.0	4.0	4
Arkion Life Sciences	USA	Private	1	8	8.0	3.0	13
CJ Cheil Jedang	Korea	Private	1	8	8.0	2.0	0
Proagny	Australia	Private	1	8	8.0	2.0	0
Snow Brand Seed	Japan	Private	1	8	8.0	8.0	26
Pancosma	Switzerland	Private	1	7	7.0	3.0	4
Alcell Technologies	Switzerland	Private	1	6	6.0	2.0	3
Camas	USA	Private	1	5	5.0	3.0	0
Rhone Poulenc Animal Nutrition	USA	Private	1	5	5.0	3.0	9

## Data Availability

Patent data are public and available on free access platforms (i.e., Es-pacenet and GooglePatents). The data presented in this study are available on request from the corresponding author.
